# Supplementary figures and images for: Odd family reunion: DNA barcoding reveals unexpected relationship between three hydrozoan species
Source: PeerJ. 2023 Apr 10;11:e15118. doi: 10.7717/peerj.15118 (PMC10100810; doi:10.7717/peerj.15118)

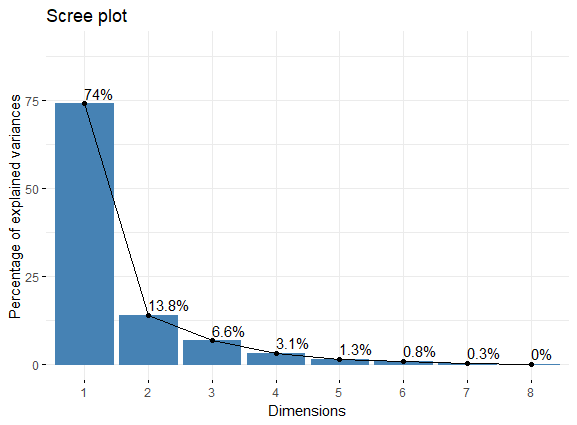

Supplement: Supplemental Information 1 — A line plot of the eigenvalues of factors or principal components used to determine the number of principal components to keep in the principal component analysis (PCA). [file peerj-11-15118-s001.png]

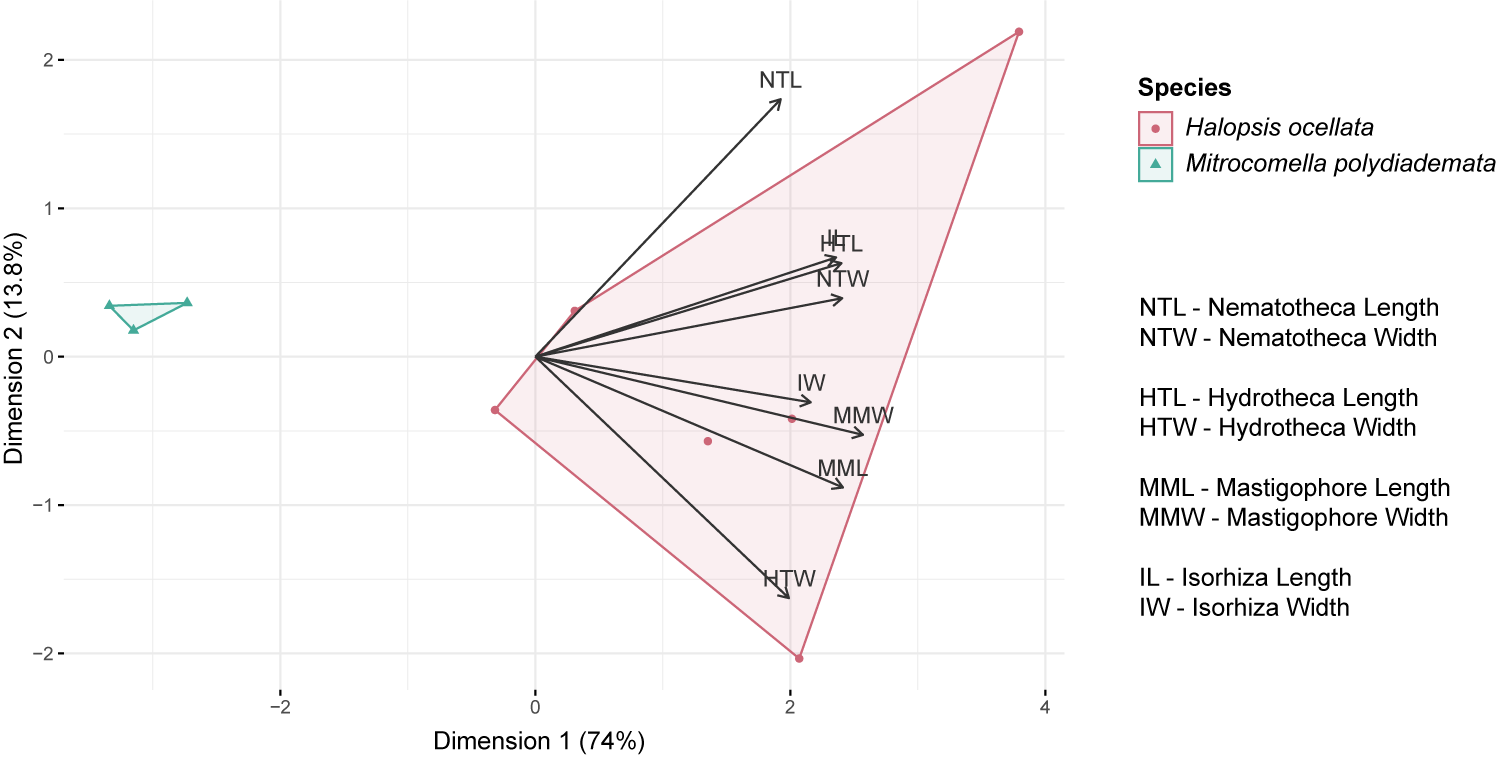

Supplement: Supplemental Information 2 — Biplot of the principal component analysis based on measurements of eight morphological characters of the newly identified polyp stage of Mitrocomella polydiademata (green) and Halopsis ocellata (red). [file peerj-11-15118-s002.png]
